# Associations of Personality Traits With Chronic Low-Grade Inflammation in a Swiss Community Sample

**DOI:** 10.3389/fpsyt.2019.00819

**Published:** 2019-11-12

**Authors:** En-Young N. Wagner, Vladeta Ajdacic-Gross, Marie-Pierre F. Strippoli, Mehdi Gholam-Rezaee, Jennifer Glaus, Caroline Vandeleur, Enrique Castelao, Peter Vollenweider, Martin Preisig, Roland von Känel

**Affiliations:** ^1^Department of Consultation-Liaison Psychiatry and Psychosomatic Medicine, University Hospital Zurich, Zurich, Switzerland; ^2^Department of BioMedical Research, Bern University Hospital, Bern, Switzerland; ^3^Department of Psychiatry, Psychotherapy and Psychosomatics, Psychiatric Hospital, University of Zurich, Zurich, Switzerland; ^4^Department of Psychiatry, Center for Research in Psychiatric Epidemiology and Psychopathology, Lausanne University Hospital and University of Lausanne, Lausanne, Switzerland; ^5^Department of Medicine, Internal Medicine, Lausanne University Hospital and University of Lausanne, Lausanne, Switzerland

**Keywords:** NEO-FFI-R, C-reactive protein, cardiovascular risk factors, inflammatory biomarkers, chronic inflammation, personality traits (big five)

## Abstract

**Objective:** Among the major dimensions of personality, high Neuroticism and low Conscientiousness have frequently been linked to worse health-related behaviors and poor health outcomes. However, studies on the association between personality traits and biomarkers of chronic low-grade inflammation reflecting increased morbidity and mortality risk are sparse; therefore, the aim of this study was to explore this association.

**Methods:** A population-based Swiss sample of 2,182 persons (40-82 years, 42% men) completed a comprehensive personality questionnaire (NEO Five-Factor Inventory-Revised). Circulating levels of inflammatory markers, including C-reactive protein, interleukin (IL)-1β, IL-6, tumor necrosis factor-α, and levels of the “cardioprotective” adipo(cyto)kine adiponectin were also determined. Analyses controlled for sociodemographic factors, traditional cardiovascular risk factors and lifetime psychiatric disorders using a validated semi-structured psychiatric interview. The role of gender as a moderator of the personality-inflammation link was additionally explored.

**Results:** Controlling for all covariates, higher Extraversion (β = 0.092, 95%CI 0.004-0.180) was positively associated with higher IL-6 levels, and higher Conscientiousness (β = -0.095, 95%CI -0.180-[-0.009]) were significantly associated with lower IL-6 levels (all p-values < 0.05). Neuroticism and Agreeableness showed no significant association with any inflammatory biomarker. The associations between personality traits and inflammatory markers were not moderated by gender.

**Conclusions:** Conscientiousness seems to be inversely related to chronic low-grade inflammation as measured by IL-6 levels, compatible with protection from the cardiovascular risk. The opposite may apply to Extraversion. Further research is needed to better understand the underlying mechanisms and their impact for health outcomes in the community.

## Introduction

Among the Big Five personality dimensions, high Neuroticism as well as low Conscientiousness have been linked to poor physical health (1), chronic illnesses (2), and mortality (3). In part, the negative health implications might be indirectly associated with these personality traits through health-related behaviors (2) like smoking, over-eating and engaging in other risky behaviors, like taking drugs (4, 5). Another pathway might be *via* direct inflammatory changes through for instance hypothalamic-pituitary axis (HPA) dysfunction, as personality traits shape the perception of and reaction to stressful situations and environments (6). In turn, it has been proposed that systemic low-grade inflammation may affect personality by depleting cognitive, emotional, and physical resources that would be needed to effectively cope with stressful situations through adaptive behaviors (7). More precisely, recent affective immunology research posits that observed health-related behaviors, which are consistent (personality) traits in a figurative sense, may mirror immune system function (8, 9) and that the immune system might control behavior rather than vice versa. In this brain – immune system interaction, low-grade inflammation becomes phenotypically apparent as a ‘behavioral immune response’ (8, 10–12). The ‘behavioral immune response’ theory (10–12) assumes that persons with rather weak immune responses exhibit more pronounced behaviors in an ecological way. Hence, a person with a less protective immune system (that is the immune system might show less activity in response to external factors) would benefit rather from harm-avoidant behaviors (e.g., greater Conscientiousness as a behavioral trait). This assumption is supported by functional genetic studies, which found an association between higher levels of Introversion with genes that carry an increased vulnerability to infections (13, 14), and by a study, in which inflammatory genes were overexpressed in individuals with higher Loneliness scores (15). Similarly, a recent study examined an association between the leukocyte gene expression and the Big Five dimensions (16). According to this study, Extraversion was associated with an increased expression of inflammatory genes and Conscientiousness was associated with a reduced expression of inflammatory genes (16).

The inflammation markers interleukin (IL)-6 and C-reactive protein (CRP) have frequently been linked to a wide range of negative health outcomes and are known to increase with age (17). The inflammatory cytokine IL-6 in particular has been shown to predict increased morbidity, including diabetes, osteoporosis, cardiovascular disease (CVD) and frailty, and also mortality (18). Researchers have primarily been interested in studies on IL-6 and on the emotion-related traits of Extraversion and Neuroticism from the Five-Factor Model (17), as these traits may be one explanation for the frequently observed elevation of IL-6 levels in depression (19, 20). In this context, two studies have investigated the association between Extraversion and IL-6, the latter personality trait being thought to be associated with an immune system that better protects against inflammation. Participants being exposed to the rhinovirus with higher scores on Extraversion-related constructs had lower levels of IL-6 in nasal lavage and fewer viral disease symptoms (21), and healthy adults showed an association between higher Extraversion scores and lower circulating IL-6 levels (22). However, in that small study (n = 103), physical activity was not controlled for as a possible confounder (22–24).

Several previous studies have examined all Five-Factor Model personality traits in the context of inflammatory biomarkers. **Table 1** shows an overview of relevant studies on these associations so far. An Italian population-based study (n = 4,923) showed an association between high Neuroticism as well as low Conscientiousness with increased IL-6 and CRP levels, even after controlling for age, gender, smoking, weight, aspirin use, and disease burden (26). In an US-population based sample (n = 1,054), there emerged an interaction between Conscientiousness and Neuroticism for circulating IL-6. Thereby, individuals with high scores in both personality traits displayed lower IL-6 levels than those with all of the other composite configurations of the Big Five dimensions (30). This interaction is likely to support the concept of “healthy neuroticism” first described by Friedmann (35), where healthy anxiety generated by Neuroticism accompanied by high Conscientiousness leads to more careful lifestyle behavior. However, it should be noted that four studies did not find any significant association between Neuroticism and IL-6 (22), perhaps due to the limited sample size (n = 103 (22); n = 91 (31) or higher age of study participants (6, 29). In a study of an US national sample of adults (n = 26,305) (7), which additionally included a meta-analysis (n = 41,605) of CRP (7) and IL-6 studies (7), Conscientiousness was inversely associated with CRP and IL-6 (7), and higher Openness was also associated with lower CRP, whereas no associations were found for Neuroticism, Extraversion and Agreeableness (7). Higher Openness (to experience) showed an inverse association with IL-6 levels in 200 persons of older age (6), as well as in the large population-based US sample mentioned above (30).

**Table 1:** Studies on the associations between the Five-Factor Model personality traits (Neuroticism, Extraversion, Openness, Agreeableness, and Conscientiousness) and inflammatory markers.

Study	Sample size (mean age ± SD)	Design^a^; personality measures& markers	Covariates	Summary of significant findings
Chapman et al. (22) 2009	103 (52.0 ± 9.0)	CD; NEO-FFI & IL-6	age, gender, ethnicity, education, depression, morbidity	activity facet of E (not E total) inversely associated with IL-6
Jonassaint et al.(25) 2010	165 (NA)	PD; NEO-PI-R & CRP	age, gender, ethnicity, education, BMI	only in African Americans (n = 94): O inversely associated with CRP
Sutin et al. (26) 2010	4,923 (39.3 ± 14.7)	CD; NEO-PI-R & CRP, IL-6	age, gender	high N and low C are associated with high IL-6 and CRP; only activity facet of E (not E total) negatively associated with IL-6
Chapman et al. (6) 2011	200 (72.8 ± 6.7)	CD; NEO-FFI & IL-6	age, gender, BMI, morbidity, smoking, alcohol, PA, diet, depression	O and C inversely associated with IL-6
Armon et al. (27) 2013	1,709 (45.6 ± 9.6)	CD; Mini-Marker & CRP, fibrinogen	age, gender, education, obesity, morbidity, medication, smoking, PA	N and E positively associated with CRP at T1 and T2, and with fibrinogen at T2; N positively associated with increased fibrinogen over four years; O negatively associated with fibrinogen at T1 and T2
Millar et al. (28) 2013	342 LDG (51.8 ± 8.0) 324 MDG (51.5 ± 8.5)	CD; EPQ & CRP, IL-6, fibrinogen ICAM-1	age, gender, education, deprivation group, BMI, smoking, alcohol intake, PA, diet, depression	LDG: N positively associated with CRP and IL-6
Mõttus et al. (29) 2013	818 (69.5 ± 0.9; 72.5 ± 0.7)	CD; NEO-FFI & CRP, IL-6	education, occupational class before retirement, childhood intelligence, morbidity, fitness, BMI, smoking, alcohol intake, PA	C inversely associated with CRP; O negatively associated with CRP; A negatively associated with fibrinogen
Turiano et al.(30) 2013	1,054 (54.6 ± 11.7)	PD; MIDI & IL-6	age, gender, ethnicity, education, BMI, smoking, alcohol, morbidity, medication	C inversely associated with IL-6; those high in both Conscientiousness and Neuroticism have low IL-6
FitzGerald et al.(31) 2014	91 (32.1 ± 10.6)	PD; NEO-FFI & IL-6	age, gender, BMI, PA	only in men: O positively associated with IL-6 only in women: A negatively associated with IL-6
Luchetti et al. (7) 2014	26,305 with studies on CRP (34, 067) or IL-6 (7, 538) (24–100 years)	meta-analysis: CD/PD; CRP, IL-6	age, gender, ethnicity, education	higher C and O associated with lower CRP; C inversely associated with IL-6
Sararoudi et al. (32) 2014	254 (51.4 ± 6.1)	CD; NEO-FFI & CRP	age, SES, BMI, dyslipidemia, fasting glucose, hypertension, smoking, diabetes	C inversely associated with CRP
Allen & Laborde (33) 2017	T1 (5, 294), T2 (3, 751); (≥50 years)	CD/PD; MIDI & CRP, fibrinogen, WBC	age, gender, ethnicity, BMI, smoking, PA	E negatively associated with CRP, fibrinogen and WBC at T1; A positively associated with CRP at T1 and T2; C negatively associated with CRP at T1, T2 and for CRP over time
Graham et al. (24) 2018	960 (57.9 ± 11.5)	CD; MIDI & CRP, IL-6, fibrinogen,	PA, age, gender, education, ethnicity, self-rated health, depression, body composition, medication, morbidity, time from personality measurement to biomarkers assessment	N inversely associated with IL-6
Schmidt et al. (34) 2018	212 (37.1 ± 12.0)	CD; NEO-PI-R & CRP, IL-2, IL-4, IL-5, IL-10, IL-12, IL-13, TNF-α, IFN- γ, GM-CSF	age, gender, smoking, BMI, morbidity, medication, time of blood sampling	N positively associated with IFN- γ, IL-5 and IL-12

a CD, cross-sectional design; PD, prospective design.

A, Agreeableness; BMI, body mass index; C, Conscientiousness; CRP, C-reactive protein; E, Extraversion; EPQ, Eysenck Personality Questionnaire; GHQ, General Health Questionnaire; GM-CSF, granulocyte macrophage colony-stimulating factor; IFN, interferon; IL, interleukin; LDG, least deprived group; MDG, most deprived group; MIDI, Midlife Development Inventory; N, Neuroticism; NA, not applicable (not tested or reported); NEO-FFI, NEO Five-Factor Inventory; NEO-PI-R, Revised NEO Personality Inventory; O, Openness; PA, physical activity; SES, socioeconomic status; T, point of time; TNF, tumor necrosis factor; WBC, white blood cell count.

The limitations of most of the previous studies are that they were restricted to only one or two inflammatory markers at a time. In this context, the inflammatory response might also differ regarding patterns of examined inflammatory biomarkers (36). This notion is supported by meta-analytic data reporting significant associations of personality traits with high sensitivity CRP, IL-1β, IL-6, and tumor necrosis factor (TNF)-α in particular. The assessment of a broader set of inflammatory biomarkers to possibly detect biomarker patterns related to distinct personality traits might be warranted to provide more insight into the psychoneuroimmunological processes relating personality to CVD risk. Therefore, guided by this objective, the present study explores the associations among the Big Five personality traits and low-grade inflammation in a large community sample, taking relevant confounding factors commonly associated with inflammation activity into account. Our study is among the first to use the Revised NEO Five-Factor Inventory-Revised (NEO-FFI-R) from the Five-Factor Model of personality, guaranteeing a comprehensive sampling of relevant personality traits. Moreover, our study included inflammatory markers that are not usually studied, including IL-1β, TNF-alpha and adiponectin, while controlling for health risk-related behaviors (including physical inactivity (23)) and performing a systematic assessment of comorbid psychiatric disorders (**Table 1**).

We primarily explored whether individuals with higher Neuroticism and/or lower Conscientiousness have higher levels of inflammatory biomarkers (CRP, IL-1β, IL-6, TNF-α) as well as lower levels of the “cardioprotective” adipo(cyto)kine adiponectin (37) than individuals with lower scores of Neuroticism and higher scores of Conscientiousness. Moreover, we explored whether individuals with higher Extraversion (22)/Openness (to experience) (6) also have lower levels of proinflammatory markers. We additionally explored the role of gender in the associations between personality traits and inflammation, as in this context, studies on gender-specific aspects are sparse (31, 38).

## Material and Methods

### Study Sample

The data of the present exploratory paper stemmed from CoLaus|PsyCoLaus (39, 40), a large ongoing multidisciplinary cohort study designed to prospectively assess the associations between mental disorders and cardiovascular risk factors (CVRFs) in the general population. Blood and plasma samples were also collected for the study of biomarkers and genetic variants.

**Figure 1:**
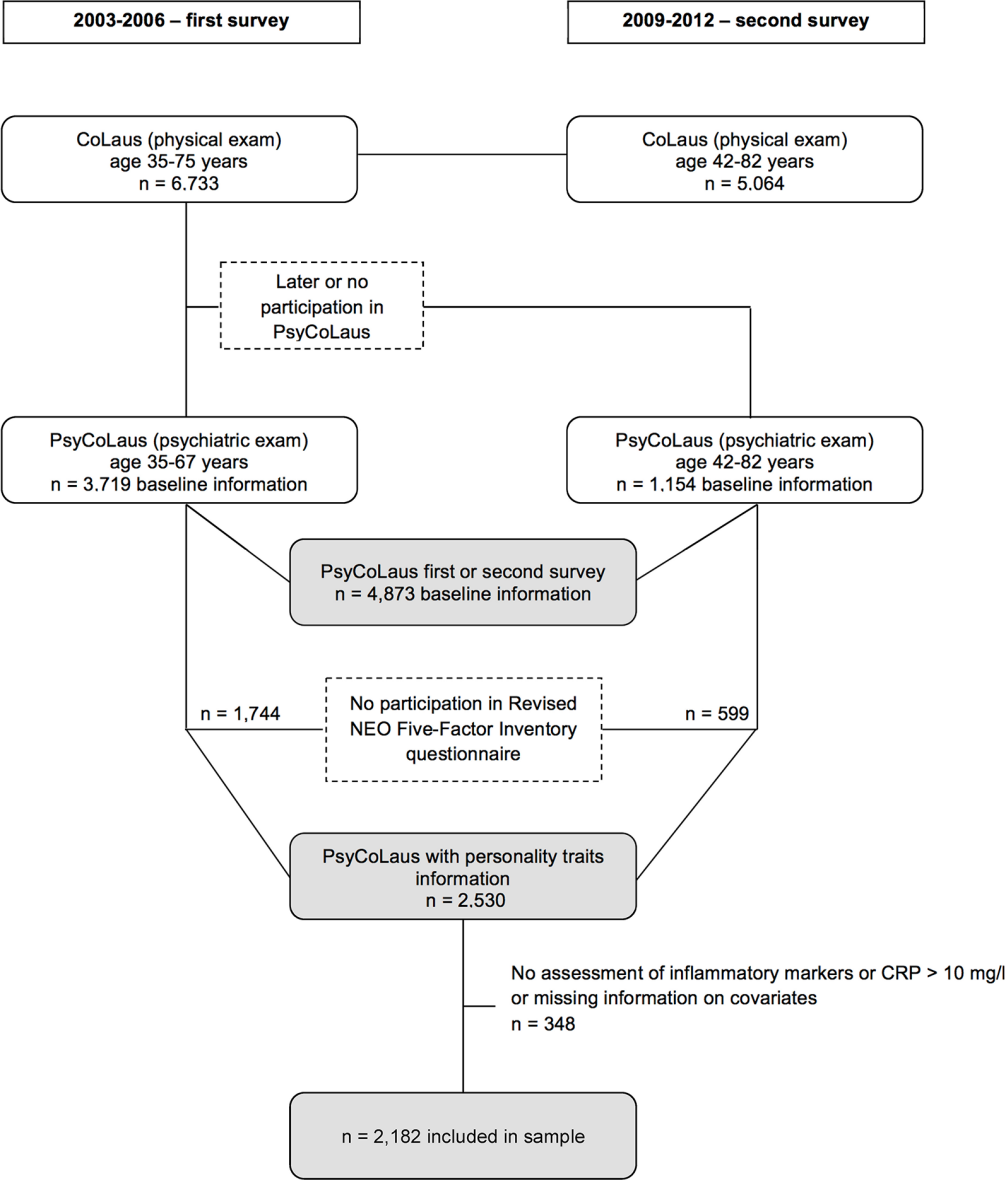
Flowchart of the CoLaus|PsyCoLaus sample for the study examining the association between personality traits and inflammatory biomarkers.

The study participants were drawn from the 35 to 75 year-old residents of Lausanne (Switzerland) according to the civil register as per January 1, 2003. The language of the study was French, as it took part in the French-speaking region of Switzerland. A final sample of 6,733 subjects agreed to participate in CoLaus after having received additional information regarding the study. The baseline somatic assessment for the CoLaus study was conducted between June 2003 and May 2006 and has been described in detail elsewhere (39). All subjects aged between 35 and 66 years were invited 1 year later to participate in PsyCoLaus, the psychiatric evaluation. In total, 67% of them were included and the final PsyCoLaus sample was comprised of 3,719 individuals who underwent both the somatic and the psychiatric exams (40). On average 5.5 years later (2009–2012) all subjects who had completed the baseline somatic assessment were invited to participate in a follow-up exam which included both a somatic and, subsequently, a psychiatric exam. Personality traits were measured with the NEO-FFI-R during the psychiatric branch of the 2nd wave of CoLaus|PsyCoLaus. A total of 2,530/4,873 (52%) participants sent back the self-rated questionnaire. The current study includes a total of 2,182 participants from these follow-up exams, aged between 40 and 82 years, who were assessed for proinflammatory markers including CRP, IL-1β, IL-6, TNF-α, and adiponectin, who completed the self-reported personality questionnaire at the follow-up psychiatric exam and who had no missing information on covariates. As values of high-sensitivity CRP > 10 mg/L are indicative of acute infection, we excluded participants with values above this threshold from the analyses. **Figure 1** shows the flowchart of the CoLaus|PsyCoLaus study sample used for the present analysis.

### Personality Assessment

Personality traits were assessed using the validated French version (41) of the NEO-FFI-R, which is a self-rating questionnaire of 60 items measuring the five major personality dimensions: 1) Neuroticism, the tendency to experience negative emotions, such as anxiety, anger, and depression; 2) Extraversion, the tendency to be sociable, warm, active, assertive, cheerful, and in search of stimulation; 3) Openness to experience, the tendency to be imaginative, creative, unconventional, emotionally and artistically sensitive; 4) Agreeableness, a dimension of interpersonal relations, characterized by altruism, trust, modesty, and cooperativeness; and 5) Conscientiousness a tendency to be organized, strong willed, persistent, reliable, and adherent to rules and ethical principles. The 60 items are answered on a 5-point Likert scale, from “strongly disagree” to “strongly agree”; some items are reverse scored to control for the effects of acquiescence. Sum scores (range 0–48) are computed for each personality dimension. The NEO-FFI-R showed good psychometric properties; Cronbach’s alpha for the internal consistency of the five factors ranged from 0.70 to 0.83 indicating good reliability (41). The mean values of NEO-FFI-R scales of our sample are comparable to those of US adults (18).

### Assessment of Inflammatory Biomarkers

**Figure 2:**
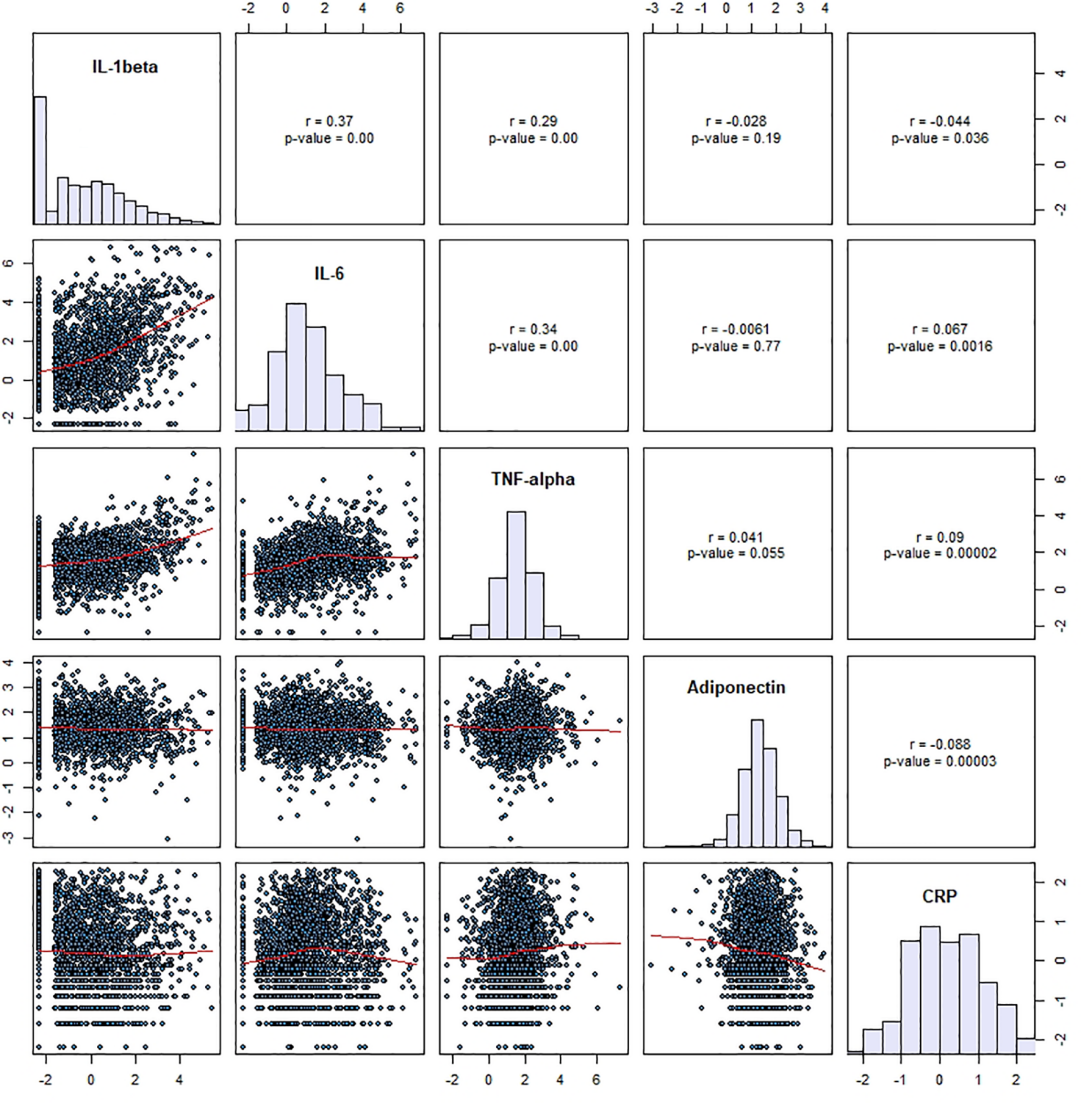
Spearman correlation coefficients between inflammatory markers in the study sample (n=2,182). IL, interleukin; TNF, tumor necrosis factor; CRP, C-reactive protein.

Morning venous blood samples (50 mL) were drawn in the fasting state and allowed to clot. Serum was preferred to plasma, as it has been shown that different anticoagulants may differentially affect absolute cytokine levels (42, 43). High-sensitivity CRP was assessed by immunoassay and latex HS (IMMULITE 1000-High, Diagnostic Products Corporation, LA, CA, USA) with maximum intra- and inter-batch coefficients of variation (CV) of 1.3% and 4.6%, respectively. Serum samples were kept at –80°C before assessment of IL-1β, IL-6, and TNF-α and sent on dry ice to the laboratory. Levels of these cytokines were measured using a multiplexed particle-based flow cytometric cytokine assay (44). This methodology yields cytokine concentrations correlating well with those obtained by other methods such as ELISA (45, 46). Milliplex kits were purchased from Millipore (Zug, Switzerland). The procedures closely followed the manufacturer’s instructions. The analysis was conducted using a conventional flow cytometer (FC500 MPL, BeckmanCoulter, Nyon, Switzerland). Good agreement between signal and cytokine was found within the assay range (R^2^ ≥ 0.99). Intra- and inter-assay CV were respectively 15% and 16.7% for IL-1β, 16.9% and 16.1% for IL-6 and 12.5% and 13.5% for TNF-α. Adiponectin was assessed by ELISA (R&D Systems, Inc, Minneapolis, USA) with a maximum inter-assay CV of 8.3% and a maximum intra-assay CV of 8.3%. For quality control, repeated measurements were conducted in 80 subjects randomly drawn from the initial sample (47). “Spearman rank correlations (n  =   80) between duplicate measurements were 0.914, 0.961, and 0.891 for IL-1β, IL-6, and TNF-α (all p < 0.001), respectively, while Lin’s correlation coefficients were 0.969, 0.971, and 0.945 and intra-class correlation coefficients were 0.970, 0.972, and 0.946 for IL-1β, IL-6, and TNF-α, respectively (all p < 0.001), indicating a good reproducibility.”(48) Overall, 2,182 subjects provided blood for the measurement of CRP, IL-1β, IL-6, TNF-α, and adiponectin. Lower limits of detection (LOD) for IL-1β, IL-6 and TNF-α were 0.2 pg/ml. Undetectable measures for IL-1β, IL-6 and TNF-α were replaced by half the LOD (i.e., 0.1 pg/ml), as was previously suggested (49, 50). For IL-6, TNF-α, and adiponectin all values were detectable. CRP showed a median of 1.20 mg/l (interquartile range (IQR): 0.60–2.40), IL-1β of 0.60 pg/ml (IQR: 0.10–2.46), IL-6 of 2.45 pg/ml (IQR: 0.96–8.08), TNF-α of 4.63 pg/ml (IQR: 2.50–8.08), and adiponectin of 3.91 mg/l (IQR: 2.50–6.16) (**Table 2**). For subsequent statistical analyses, inflammatory measures were log10-transformed to normalize distributions. Spearman’s correlation coefficients between the different inflammatory markers were not high (**Figure 2**).

**Table 2:** Characteristics of the study participants (n = 2,182).

Variable	Mean ± SD (range) or percentage value
Age, years	58.4 ± 10.2 (40.3–81.6)
Gender (male/female), %	41.9/58.1
Socioeconomic status, % High/Middle/Low/Very low	18.7/28.1/28.7/24.5
Systolic blood pressure, mm Hg	125.7 ± 17.9 (79.5–210.0)
Glucose, fasting state, mmol/L	5.8 ± 1.0 (1.5–23.9)
Body mass index, kg/m²	25.7 ± 4.3 (14.2– 47.2)
LDL/HDL-cholesterol ratio	2.2 ± 0.9 (0.3–6.7)
Smoking status, % Never Former/Current	40.7 59.3
Physical inactivity, %	26.2
**Psychiatric disorders, DSM-IV, %**	
Mixed disorders	4.4
Depressive disorders	17.1
**Personality traits (range 0–48)**	Mean ± SD (range)
Neuroticism	18.2 ± 7.6 (0–45)
Extraversion	27.8 ± 6.1 (3–45)
Openness	29.6 ± 5.9 (9–48)
Agreeableness	33.5 ± 5.1 (12–48)
Conscientiousness	35.1 ± 5.6 (7–48)
**Inflammatory measures**	Median (interquartile range)
C-reactive protein, mg/l	1.20 (0.60–2.40)
Interleukin-1β, pg/ml	0.60 (0.10–2.46)
Interleukin-6, pg/ml	2.45 (0.96–8.08)
Tumor necrosis factor-α, pg/ml	4.63 (2.50–8.08)
Adiponectin, mg/l	3.91 (2.50–6.16)

SD, standard deviation; HDL, high-density lipoprotein; LDL, low-density lipoprotein.

### Assessment of Covariates

We controlled for a number of variables that are known to have an effect on circulating levels of proinflammatory biomarkers: socio-demographic characteristics [age, sex, socioeconomic status (SES)], cardio-metabolic risk factors [blood pressure (triplicate measure on the left arm after at least a 10-min rest in the seated position), body mass index (BMI) derived from measured weight and height, glucose fasting state and low-density lipoprotein (LDL)/high-density lipoprotein (HDL) ratio], behavioral factors (smoking and physical inactivity) as well as psychiatric disorders. SES was assessed using the Hollingshead scale (49). Smoking was categorized into former, current and non-smokers. Physical inactivity was defined as less than 20 min of activity twice a week, and categorized as a binary variable. The levels of glucose, LDL-cholesterol, and HDL-cholesterol were drawn from venous blood samples for each participant after an overnight fast. Diagnostic information on psychiatric disorders was collected using the semi-structured Diagnostic Interview for Genetic Studies (DIGS) (50). The DIGS was developed by the National Institute of Mental Health (NIMH) Molecular Genetics Initiative to obtain a more precise assessment of phenotypes through a wide spectrum of DSM-IV Axis-I criteria. Psychiatric diagnoses were assigned according to the DSM-IV. We used the French translation of the DIGS (48) with excellent inter-rater reliability in terms of kappa and Yule’s Y coefficients for major mood and psychotic disorders (51), as well as for substance use disorders (52), and the 6-week test-retest reliability, which was somewhat lower, was still in the fair to good ranges (51, 52). In order to adequately assess anxiety disorders, the DIGS was completed with the anxiety sections of the French version (53) of the Schedule for Affective Disorders and Schizophrenia - Lifetime and Anxiety disorder version (SADS-LA) (54) which showed satisfactory reliability (53). In our own clinical family study using the French version of the DIGS, the inter-rater reliability for specific anxiety disorders was very good, whereas the 6-week test-retest reliability estimates were in the fair or good ranges (55). Interviewers were required to be master’s level psychologists, who were trained over a 1 to 2-month period. Their training included rating tapes and supervised co-ratings. To provide ongoing supervision throughout the study, each interview and diagnostic assignment was reviewed by an experienced senior psychologist. The psychiatric diagnoses were cumulative over the lifetime until the moment of the follow-up exam.

### Statistical Analysis

Associations between each of the five personality traits and CRP, IL-1β, IL-6, TNF-α, adiponectin outcome levels were determined using multiple linear regression models, separately for each outcome variable. Statistical significance was considered at p < 0.05. Data are presented as means ± standard deviation and range or percentage values, and inflammatory measures are given as medians with interquartile range. For the associations between each of the five personality traits (all five entered simultaneously into each model) and inflammatory markers, three models of increasing complexity were computed: in model 1 we adjusted for sociodemographic factors (age, gender, SES) only, in model 2, we additionally adjusted for cardio-metabolic risk factors (systolic blood pressure, fasting glucose, BMI, total LDL/HDL-cholesterol ratio) and behavioral factors (smoking, physical inactivity), and finally, in model 3, we additionally adjusted for lifetime DSM-IV psychiatric disorders after having classified them using latent class analysis (see below). Additionally, we examined the potential impact of gender in terms of an interaction on the association between personality traits and inflammatory biomarkers by performing the analyses with gender*personality trait interaction terms. Given the large number of models including interaction terms, to avoid the impact of multiple comparison on interaction terms, we determined the cut-off for a significant interaction at p < 0.01. All variance inflation factors in regression models were below 2.5, indicating that there was no concern regarding collinearity.

We used the IBM^®^ SPSS^®^ 23.0 statistical software package (IBM Corporation, New York, USA), and the Statistical Analysis System, version 9.4 for Windows (SAS Institute Inc., Cary, NC, USA).

**Figure 3:**
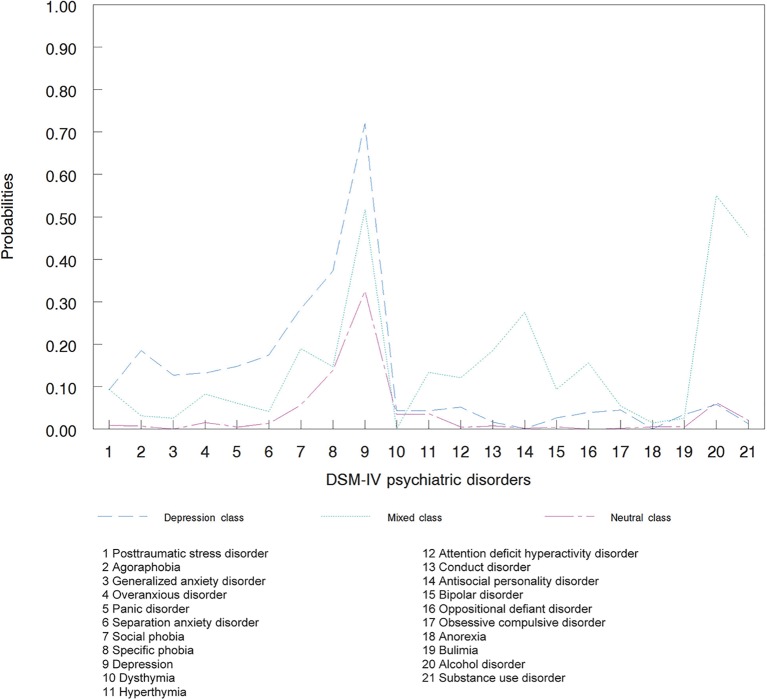
Latent classes of DSM-IV psychiatric disorders (n = 2,182).

The main statistical analysis, based on cross-sectional data, was preceded by a latent class analysis (LCA) of 21 neurodevelopmental and common mental disorders among 2,182 individuals in order to identify distinct patterns of psychiatric comorbidity (DSM IV-disorders) and adjust for them in the regression models described above. LCA is a person-centered approach (56) which detects homogenous groups of individuals, in this instance, based on similar diagnostic patterns. Based on the Akaike information criterion and the Bayesian information criterion, the LCA solution with three classes was chosen. The classes reflected the well known differentiation between externalizing and internalizing disorders. **Figure 3** shows the three latent classes of DSM-IV psychiatric disorders. Depressive disorders displayed higher probabilities in the first class (n = 95), whereas the second class comprised higher probabilities of mixed disorders (n = 374). The third class (n = 1,713) was the neutral class, comprising subjects with no or sporadic mental disorders. The LCA was conducted using Latent GOLD^®^ 5.1 (Statistical Innovations, MA, USA).

## Results

### Sample Description

**Table 1** shows the demographic, metabolic, and personality characteristics of the sample. Participants (n = 2,182) were between 40 and 82 years of age (mean ± SD = 58.4 ± 10.2) and 42% were males. Concerning the cardio-metabolic risk factors, quite a sizeable percentage of participants were former or current smokers (59%), they had a wide range of mean systolic blood pressure (79–210 mm Hg; mean ± SD = 125.7 ± 17.9 mm Hg) and of BMI (14.2–54.2 kg/m^2^; mean ± SD = 25.7 ± 4.3 kg/m^2^; **Table 1**). Only 26.2% reported to be physically inactive. The study sample had relatively low Neuroticism scores [mean ± SD (range): 18.2 ± 7.6 (0–5)] and high Conscientiousness scores [35.1 ± 5.6 (7–48)].

A huge attrition might reduce the generalizability of the findings. To address this issue, we are able to compare the final sample (n = 2,182) with the original sample (n = 6,733) by age, gender and the General Health Questionnaire (GHQ-12). GHQ-12 is the unique measurement of psychopathology screening available for CoLaus participants at baseline. The higher the GHQ-12 score, the more severe the psychological distress. Among 6,733 participants to CoLaus baseline, 5,807 filled in the GHQ-12 questionnaire. GHQ-12 was available for 1,940/2,182 participants in the study sample. The comparison between this group (n = 1,940) and the remaining participants of the original sample (n = 3,867) with GHQ-12 scores on age, gender, and GHQ-12 revealed significantly more men (57.9/49.7%; p < 0.001) and lower GHQ-12-scores (mean ± SD: 1.49 ± 2.28/1.75 ± 2.66; p < 0.001) in the study sample.

### Associations Between Personality Traits and Inflammatory Markers

In the fully adjusted model 3 (**Table 3**), significant associations emerged for three personality traits and IL-6 levels, although not with any other inflammatory marker. Whereas higher Extraversion was associated with higher IL-6, higher Openness, and higher Conscientiousness were both associated with lower IL-6. There were no significant interactions between gender and personality traits for any inflammatory marker (all p-values > 0.01). This implies similar relationships between personality traits and inflammatory markers in both men and women.

**Table 3:** Associations between personality traits and inflammatory markers or adipokine concentrations (n = 2,182).

	Model 1		Model 2		Model 3	
	β	95CI	β	95CI	β	95CI
**Interleukin-1β**, pg/ml						
Neuroticism	0.015	(–0.073, 0.104)	0.016	(–0.073,0.105)	–0.002	(–0.094,0.090)
Extraversion	0.043	(–0.045, 0.132)	0.046	(–0.044,0.135)	0.047	(–0.042,0.137)
Openness	–0.059	(–0.139, 0.021)	–0.058	(–0.138,0.023)	–0.066	(–0.147,0.015)
Agreeableness	–0.030	(–0.112, 0.051)	–0.032	(–0.114,0.049)	–0.030	(–0.112,0.052)
Conscientiousness	–0.036	(–0.122, 0.051)	–0.036	(–0.123,0.051)	–0.034	(–0.121,0.053)
**Interleukin-6**, pg/ml						
Neuroticism	0.035	(–0.053, 0.122)	0.038	(–0.050,0.126)	0.019	(–0.072,0.110)
Extraversion	0.099*	**(0.011, 0.187)**	0.089*	**(0.001,0.177)**	0.091*	**(0.003,0.179)**
Openness	–0.066	(–0.145, 0.013)	–0.072	(–0.151,0.007)	–0.080*	(–**0.160,0.000)**
Agreeableness	0.064	(–0.016, 0.145)	0.072	(–0.009,0.152)	0.074	(–0.007,0.154)
Conscientiousness	–0.100*	(–**0.185,-0.014)**	–0.095*	(–**0.181,-0.010)**	–0.094*	(–**0.180,-0.008)**
**Tumor necrosis factor-α**, pg/ml						
Neuroticism	–0.003	(–0.055, 0.049)	–0.002	(–0.054,0.050)	–0.002	(–0.056,0.051)
Extraversion	0.012	(–0.040, 0.064)	0.005	(–0.047,0.057)	0.004	(–0.048,0.057)
Openness	–0.036	(–0.083, 0.011)	–0.038	(–0.085,0.009)	–0.039	(–0.086,0.009)
Agreeableness	–0.025	(–0.073, 0.023)	–0.020	(–0.068,0.028)	–0.020	(–0.068,0.028)
Conscientiousness	–0.011	(–0.061, 0.040)	–0.006	(–0.057,0.045)	–0.005	(–0.056,0.046)
**High-sensitivity C-reactive protein**, mg/l						
Neuroticism	0.001	(–0.044, 0.047)	0.000	(–0.041,0.040)	–0.005	(–0.047,0.037)
Extraversion	0.018	(–0.028, 0.064)	0.008	(–0.032,0.049)	0.007	(–0.033,0.048)
Openness	–0.030	(–0.071,0.011)	–0.018	(–0.055,0.019)	–0.022	(–0.059,0.015)
Agreeableness	–0.009	(–0.051,0.032)	–0.013	(–0.050,0.024)	–0.011	(–0.048,0.026)
Conscientiousness	–0.009	(–0.053,0.036)	0.017	(–0.023,0.056)	0.021	(–0.019,0.060)
**Adiponectin**, mg/ml						
Neuroticism	–0.016	(–0.049, 0.017)	–0.015	(–0.047,0.017)	–0.021	(–0.054,0.012)
Extraversion	–0.029	(–0.062, 0.005)	–0.025	(–0.057,0.007)	–0.024	(–0.056,0.008)
Openness	–0.019	(–0.048, 0.011)	–0.019	(–0.048,0.010)	–0.021	(–0.050,0.008)
Agreeableness	0.028	(–0.002, 0.059)	0.026	(–0.003,0.056)	0.026	(–0.003,0.056)
Conscientiousness	0.010	(–0.022, 0.042)	0.001	(–0.030,0.033)	0.001	(–0.031,0.032)

*p <0.05.

95CI; 95% confidence interval.

Model 1: Linear regression model adjusted for age, gender, socio-economic status.

Model 2: Model 1 + adjustment for systolic blood pressure, glucose, body mass index, LDL/HDL cholesterol ratio, smoking status, physical inactivity.

Model 3: Model 2 + adjustment for latent classes of DSM-IV disorders. The bolded values (text) are statistical significant.

## Discussion

In this Swiss community sample of 2,182 adult individuals, the main finding was that Extraversion, Openness, and Conscientiousness seemed to be significantly associated with IL-6 levels. The associations between personality traits and inflammatory markers were not moderated by gender, and such analyses have not systematically been performed in previous studies (38). Moreover, the association between personality traits and inflammation was maintained after adjustment for mental disorders (Model 2 vs. Model 3), which is in line with previous studies that have shown that correlations between personality traits (other than Neuroticism) with mental disorders/symptoms are rather modest (57, 58).

Previous studies (**Table 1**) examining an association between Extraversion and IL-6 showed a different approach in terms of adjustments for confounding factors. For instance, physical inactivity (7, 22, 26), SES (6, 26, 31, 33), gender (32), and psychiatric morbidity (30), including depression and alcohol consumption, were not controlled in these studies. Also, previous studies examined specific populations (elderly persons (29), deprived individuals (28)), which might explain different study results. Among those studies, the studies by Millar et al. (28) and by Graham et al. (24) seem to show the greatest similarities in their study design and sample with our study. However, other personality assessment instruments were used in both these studies (**Table 1**), which might also have had an impact on different study results. To our knowledge, this study is among the first to suggest a positive association between higher Extraversion and increased low-grade inflammation, i.e., elevated IL-6, when controlling for physical inactivity, as has been suggested in previous studies (22, 24). Also, a small study (n = 58), which assessed Extraversion using the Karolinska Scale of Personality (KSP), found that its subscale of Impulsivity determined the significant correlation with higher IL-6 levels among suicide attempters (59), but physical activity was also not taken into account. However, we feel reluctant to reason forward, as it is not clear to what extent Extraversion assessed with the KSP in that study is comparable to Extraversion assessed with the NEO-FFI-R. High Extraversion might imply that someone is more of a sensation-seeker and therefore has more interpersonal difficulties, which have been associated with an increased sensitivity to hyperinflammatory responses (60). In this context, a dysfunctional HPA axis has been suggested to play a potential role in the link between trait components and inflammatory activity; however, further mechanistic studies are needed to examine to what extent this might apply to particular personality traits. For instance, higher Neuroticism in women but lower Extraversion in men have both been associated with blunted cortisol responses to stress (61), which, in turn, may result in greater inflammatory reactivity evidenced by elevated IL-6 levels (62).

Our data also suggest an inverse association between Openness (to experience) and IL-6 levels. This finding is congruent with previous studies in which the association of Openness with IL-6 was investigated in diverse populations over different periods of time (6, 29, 30). However, the association between Openness and IL-6 in our study might be spurious as it only became significant when the model was adjusted for DSM-IV disorders, and with a p-value of p = 0.049, the confidence interval touches 0. Interestingly, such an association was not revealed in a recent meta-analysis (7). However, there was considerable heterogeneity between studies included in that meta-analysis in terms of study designs and statistical approaches so that this result should be interpreted with caution (7).

The suggested significant association between higher Conscientiousness and decreased IL-6 levels was in the expected direction based on the results of a previous meta-analysis (7). Moreover, our findings of an association between higher Conscientiousness and lower IL-6 concur with studies on individuals with lower levels of this personality trait and poor physical health (1), chronic illnesses (2), and mortality (3). The finding also concurs with four previous studies (26, 29, 32, 33), whereas five other studies did not find a significant association between Conscientiousness and IL-6 (24, 25, 27, 28, 33). Based on its comprehensive approach as discussed above, our study may support the interpretation that there could be a modest inverse association between Conscientiousness and IL-6.

There was no significant association of Neuroticism with IL-6 levels in our study sample. Also, the majority of the studies so far have found no association between Neuroticism and IL-6, both in a large study (n = 26,305) (7) that additionally had performed a meta-analysis on IL-6 (n = 7,538) (7), and in five other studies (6, 7, 22, 31). Instead, another study found an interaction between Neuroticism and Conscientiousness in that participants with high scores in both these personality traits had lower IL-6 levels than those with the other combinations of high and low scores for Neuroticism and Conscientiousness (30). Unfortunately, we were not able to replicate these analyses due to the low number of participants displaying this combination of personality traits. Compared to studies which found a significant association between Neuroticism and IL-6 (26, 30) or CRP (26), the average Neuroticism scores in our study were relatively low [mean ± SD (range) = 18.2 ± 7.6 (0–45)], such that our analysis may have yielded a rather conservative estimate. Further, two studies found a positive (26, 28) association, and one study a negative (24) association between Neuroticism and IL-6. These results replicate and extend the existent body of literature in the context of personality characteristics, the results for an association between Extraversion and IL-6 being novel, by taking health behaviors as potential confounders into account, for which particular personality traits are at risk (2, 4, 5).

Contrary to our expectation, there were no significant associations between any personality trait and CRP in our study. Our finding concurs with two recent studies (24, 34), whereas seven other studies showed an association between Neuroticism and/or Conscientiousness and CRP. The differing study results might be explained by a wider age range of participants (e.g., 14–82 years in Sutin et al.) than in our study (40–82 years), implying that this association might become more apparent when assessed over the entire age range, or taking into account other factors discussed above.

Similar to previous studies on the association between personality and inflammation, IL-6 showed robust significance in our study, whereas the other inflammatory markers did not. As opposed to the other inflammatory markers, research on IL-6 appears to be a more comprehensive field of psychoneuroimmunology perhaps due to its greater role in this context (63). This might also be due to its tasks in various biodynamic processes including stress, physical activity, and circadian rhythms (64), but also to its significance in the risk for CHD (63, 65–70). Thereby, long-term increased IL-6 levels have been classified as strong as traditional CVRFs (66); furthermore, human genetic studies identified causal IL-6 signaling in CHD (70, 71). Given the role of IL-6 in biobehavioral research taken together, it seems plausible that personality as a bio-psycho-dynamic entity might evidently be reflected by this cytokine. Indeed, genetic factors, environmental circumstances, and illness/adversity may all contribute to personality (change). For instance, IL-6 levels raised in individuals *in vivo* led to increased negative affectivity and low performance scores on memory tasks (72).

The clinical impact of a personality trait (e.g., Conscientiousness) may be exemplified indirectly by studies which examined an association between inflammatory biomarkers and the cardiovascular risk. For instance, according to a recent meta-analysis of prospective population-based studies, a one standard deviation increased baseline level for log_e_ IL-6 predicted a 25% adjusted higher relative risk of non-fatal myocardial infarction or coronary heart disease (CHD) mortality (65). Based on this meta-analytic data, and using baseline IL-6 levels in our study sample, a decrease of 41 units in the Conscientiousness score would be required in the fully adjusted model 3 to increase the relative risk of non-fatal myocardial infarction or CHD mortality by 10% (calculation not shown). Interestingly enough, as Conscientiousness scores ranged from 7 to 48 in our study sample (**Table 2**), this would also mean that individuals with the lowest Conscientiousness scores had a 10% higher relative risk of incident CHD than those with the highest Conscientiousness score. These calculations imply that the relationship between Conscientiousness and IL-6 levels could be of clinical relevance.

The major strength of this population-based study was the combination of a thorough biological evaluation comprising objectively measured health-related risk factors with a comprehensive psychiatric assessment using a face-to-face interview conducted by master’s level psychologists. Our study is among the first to use the NEO-FFI-R from the Five-Factor Model of personality, guaranteeing a comprehensive sampling of relevant personality traits. Moreover, our study included inflammatory markers that are not usually studied, including IL-1β, TNF-alpha, and adiponectin, while controlling for health risk-related behaviors (including physical inactivity) and performing a systematic assessment of comorbid psychiatric disorders. The study was designed to better understand the relationship between psychiatric disorders and CVD (39) as well as its underlying mechanisms, including the role of chronic low-grade inflammation as one of these potential mechanisms. Enhanced inflammation is a key process in atherosclerosis (73, 74). Biopsychosocial research also identified personality traits as potential risk factors affecting the initiation and progression of atherothrombotic diseases (75, 76). In this context, the results of the current study may support the assumption that chronic low-grade inflammation could independently link low Conscientiousness and high Extraversion with CVD risk (35). Moreover, our study is among the first to examine the link between the NEO-FFI-R personality traits and IL-1β, TNF-α, and/or adiponectin in a large population-based sample, whereby showing no significant associations with these personality dimensions, which findings must be replicated in future studies.

The findings of our study should be interpreted with respect to four notable limitations. First, the cross-sectional design of our study does not allow causal interferences on the direction of the relationship between personality traits and (chronic) low-grade inflammation. Furthermore, it may be that the interaction between personality and inflammation is moderated by a third parameter, including genetic polymorphisms. Second, we were not able to apply our statistical models to the full age range, as the participants from this study were between 40 and 82 years of age; therefore, our findings cannot be transferred to all age groups. Also, these universally accepted personality traits seem to exhibit distinct inflammatory patterns among our middle aged and older adults, as the significant relationships were also independent of the four other traits. Third, the risk of loss to 5-year follow-up may be particularly driven by individuals with personality traits who struggle to adhere to activities over a longer period of time, e.g., those high in Neuroticism. Hence, our sample might include less individuals with higher scores in Neuroticism which might also explain why our sample did not show an association between Neuroticism and inflammatory biomarkers as reported in other studies. Fourth, participants in the final study sample had lower levels of psychological distress compared to the remaining participants of the original sample. However, this might explain why our “healthier” sample did not show an association between Neuroticism and inflammatory biomarkers as reported in other studies. Fifth, given the large set of covariates in multivariate regression models, collinearity could have been a potential problem. However, to prevent such an effect, redundant variables were removed and similar variables were aggregated. We observed no trace of any multicollinearity in adjusted models.

To sum up, our study has suggested independent modest associations between high Extraversion and low Conscientiousness with high IL-6. These findings suggest that previous studies showing poor health outcomes to be linked to particular personality traits could partially be explained by inflammatory activity. Low-grade inflammation might contribute to vascular pathologies, mainly atherothrombotic CVD, but also other adverse health outcomes over time. However, this hypothesis and their clinical relevance in the community need to be confirmed in longitudinal studies with CVD and morbidity/ mortality as clinical outcomes. Performing such studies might additionally offer the possibility to better elucidate the components (facets) of the different personality traits which are specifically associated with chronic low-grade inflammation.

## Data Availability Statement

All outputs generated for this study are included in the article/ supplementary material.

## Ethics Statement

The Institutional Ethics Committee of the University of Lausanne approved the CoLaus and subsequently the PsyCoLaus study. All participants signed a written informed consent in accordance with the Declaration of Helsinki after having received a detailed description of the goal and funding of the study.

## Author Contributions

Conceived and designed the experiments: E-YW, MP, PV, and RK. Performed the experiments: E-YW and RK. Analyzed the data: E-YW, VA-G, M-PS, MG-R and RK. Contributed reagents/materials/analysis tools: E-YW, VA-G, MG-R, EC, JG, MP, PV, and RK. Wrote the paper: E-YW, VA-G, M-PS, CV, JG, and RK.

## Funding

PV and MP received two unrestricted grants from GlaxoSmithKline, Verona, Italy.

The CoLaus|PsyCoLaus study was and is supported by research grants from the Faculty of Biology and Medicine of Lausanne (Switzerland) and the Swiss National Science Foundation (Switzerland) to MP (grants 3200B0-105993, 3200B0-118308, 33CSCO-122661, 33CS30-139468, and 33CS30-148401). The funders had no role in the design of the study; the collection, management, analysis, and interpretation of the data; the preparation, review, or approval of the manuscript; or the decision to submit the manuscript for publication.

## Conflict of Interest

The authors declare that the research was conducted in the absence of any commercial or financial relationships that could be construed as a potential conflict of interest.
